# Supplementary material for: The Construction and Exploration of a Comprehensive MicroRNA Centered Regulatory Network in Foxtail Millet (Setaria italica L.)
Source: Front Plant Sci. 2022 May 6;13:848474. doi: 10.3389/fpls.2022.848474 (PMC9121102; doi:10.3389/fpls.2022.848474)

T=Seita.2G033100.1_Q=Sit−miR396c_S=3425

category=2_p=0.97283142649702

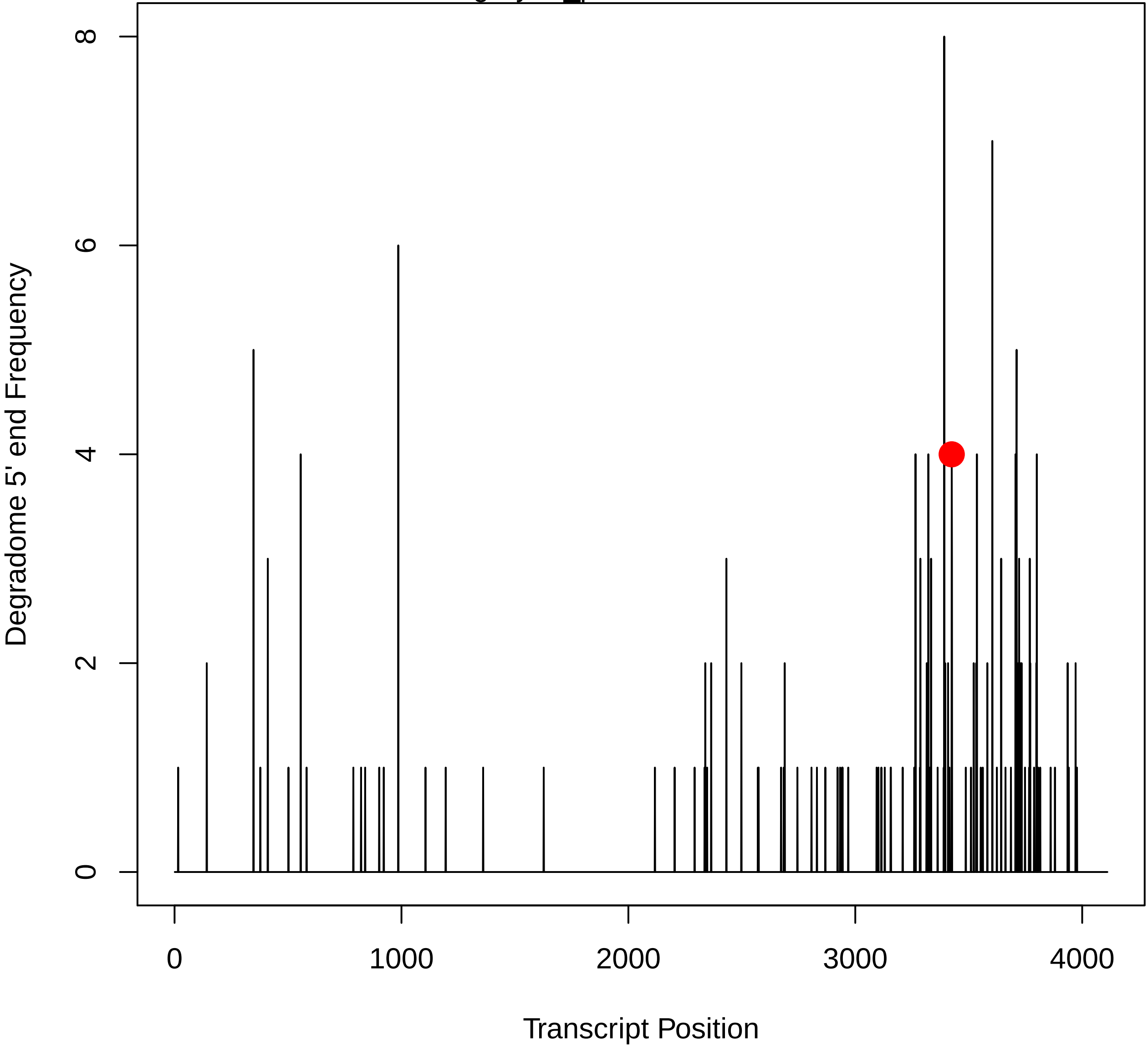

Supplement: Supplementary file 1 [file Data_Sheet_1.zip › Sit-miR396c_Seita.2G033100.1_3425_TPlot.pdf]
